# DNA Methylation in Huntington’s Disease

**DOI:** 10.3390/ijms222312736

**Published:** 2021-11-25

**Authors:** Nóra Zsindely, Fruzsina Siági, László Bodai

**Affiliations:** 1 Department of Genetics, Faculty of Science and Informatics, University of Szeged, Közép fasor 52, H-6726 Szeged, Hungary; zsindely@bio.u-szeged.hu; 2 Department of Biochemistry and Molecular Biology, Faculty of Science and Informatics, University of Szeged, Közép fasor 52, H-6726 Szeged, Hungary; siagifru@gmail.com; 3 Doctoral School in Biology, Faculty of Science and Informatics, University of Szeged, H-6726 Szeged, Hungary

**Keywords:** Huntington’s disease, neurodegeneration, DNA methylation, transcriptional dysregulation, CpG methylation, 5-methylcytosine, DNA methyltransferase

## Abstract

Methylation of cytosine in CpG dinucleotides is the major DNA modification in mammalian cells that is a key component of stable epigenetic marks. This modification, which on the one hand is reversible, while on the other hand, can be maintained through successive rounds of replication plays roles in gene regulation, genome maintenance, transgenerational epigenetic inheritance, and imprinting. Disturbed DNA methylation contributes to a wide array of human diseases from single-gene disorders to sporadic metabolic diseases or cancer. DNA methylation was also shown to affect several neurodegenerative disorders, including Huntington’s disease (HD), a fatal, monogenic inherited disease. HD is caused by a polyglutamine repeat expansion in the Huntingtin protein that brings about a multifaceted pathogenesis affecting several cellular processes. Research of the last decade found complex, genome-wide DNA methylation changes in HD pathogenesis that modulate transcriptional activity and genome stability. This article reviews current evidence that sheds light on the role of DNA methylation in HD.

## 1. Introduction

Huntington’s disease (HD) is a monogenic dominant neurodegenerative disorder that mainly affects the striatum but other areas of the brain are also impacted. Its symptoms, which involve motor, cognitive and psychiatric disturbances first manifest in ~40 years old adults and lead to the patient’s death after ~15–20 years long progression [1]. The disease is caused by a dominant gain of function in the *huntingtin* (*HTT*, *IT15*) gene located in the cytogenetic position 4p16.3 [2]. *HTT* is a large gene that spans a 170 kb genomic region, contains 67 exons, and encodes a 3144 amino acid long, 348 kDa Huntingtin (Htt) protein. Htt contains 36 HEAT (Huntingtin, elongation factor 3, protein phosphatase 2A, TOR1) repeats organized into 5 blocks, an N-terminal nuclear localization sequence (NLS), and a C-terminal leucine-rich nuclear export signal (NES). In the first exon of *HTT*, there is a polymorphic, unstable CAG trinucleotide repeat sequence that is translated to a polyglutamine (polyQ) repeat close to the N-terminus of Htt. The disease-causing mutation is an increase in the repeat number of the CAG repeat that results in an elongated polyQ domain in the mutant Htt (mHtt) protein. While repeats with less than 36 glutamines are non-pathogenic, repeats with more than 39 glutamines cause disease with full penetrance (alleles with 36–39 CAG repeats have reduced penetrance) [1,3]. Although *HTT* is ubiquitously expressed the disorder is characterized by selective loss of neurons that mainly affect the medium spiny neurons (MSN) of the striatum. mHtt is prone to aggregation and the polyQ domain also mediates improper interactions with important cellular proteins. The cellular pathogenesis of HD, which involves the disturbance of several molecular pathways and cellular processes, is mainly based on these aberrant protein interactions. One of the pathogenetic processes that precede overt symptoms is transcriptional dysregulation [3,4,5]. Mutant Huntingtin modulates the level or activity of specific transcriptional factors, such as Elk-1, SMAD3, Sp1, or Twist1 [4,6,7,8], and also disturbs transcriptional control by affecting processes regulating the structure and accessibility of chromatin, including histone methylation, histone acetylation and DNA methylation [9,10,11]. The potential therapeutical importance of epigenetic effects in HD pathology is underlined by studies showing that symptoms can be mitigated by histone deacetylase (HDAC), histone demethylase, and DNA methyltransferase (DNMT) inhibitors in HD models [12,13,14]. In this review, we overview the body of knowledge accumulated about altered DNA methylation and its implications in the pathology of Huntington’s disease.

## 2. DNA Methylation and Its Role in Transcriptional Regulation

DNA methylation is an epigenetic mechanism that in mammals is essential for tissue-specific gene expression regulation, silencing of retroviral elements/repetitive DNA sequences, genomic imprinting, and X chromosome inactivation. DNA methylation occurs predominantly on cytosine bases preceding guanine in the DNA sequence, which are called CpG dinucleotides or CpG sites. CpG sites are underrepresented in the genome except in short (~few hundred to ~1000 bp) genomic regions, the so-called CpG islands (CGIs) [15]. The majority (~70%) of human gene promoters reside next to CGIs which are usually not methylated, while non-CGI CpG sites across the genome are heavily methylated. CGI methylation results in stable silencing of gene expression and has an important function in transcription regulation during development and differentiation and in the maintenance of genomic imprinting. The main role of CpG methylation in the intergenic regions is to repress transposable elements and repeat regions and is important for genome stability [16].

**Figure 1:**
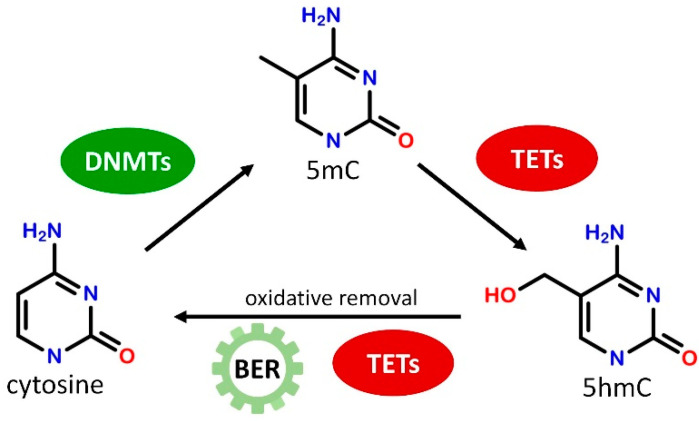
Schematic illustration of the generation and removal of 5-methylcytosine (5mC) marks. Cytosines in the CpG context can be methylated by DNA methyltransferases (DNMTs). 5mC can be converted to 5-hydroxymethylcytosine (5hmC) by Ten-Eleven Translocation (TET) dioxygenases, and then can be removed by the multistep action of TET enzymes and base excision repair (BER).

DNA methylation is established by DNA methyltransferase enzymes (DNMTs) that catalyze the transfer of a methyl group to the C5 position of cytosine residue from S-adenosyl methionine (SAM) resulting in 5-methylcytosine (5mC, Figure 1). DNMT1 is responsible for maintaining CpG methylation patterns during DNA replication by copying the methylation pattern from the parental DNA strand to the newly synthetized strand in hemimethylated DNA [17]. DNMT3a and DNMT3b, on the other hand, are known as *de novo* methyltransferases as they can establish new methylation patterns on unmodified DNA [18]. Both maintenance and *de novo* methyltransferases have crucial importance in cellular function, DNMT1 has a critical role in dividing cells as well as in cellular differentiation while DNMT3a and DNMT3b are required for normal cellular differentiation and early development. DNMT1 and DNMT3a alike are expressed at substantial levels in post-mitotic neurons indicating that proper DNA methylation may play a significant regulatory function in the adult brain also [19]. DNA methylation marks can be eliminated by diverse enzymatic mechanisms, but no enzymes are known to date to directly remove the methyl group from 5mC. Instead, during active DNA demethylation, a series of consecutive enzymatic reactions are necessary to convert 5mC to cytosine. 5mC can be modified by deamination and/or oxidation reactions to intermediate products resulting in a mismatch in DNA base pairing that can be recognized by the base excision repair (BER) pathway and replaced by cytosine [20]. In the well-characterized oxidative demethylation of DNA Ten-Eleven Translocation (TET) dioxygenase enzymes, including TET1, TET2, and TET3, oxidize 5mC in consecutive steps to generate 5-hydroxymethylcytosine (5hmC), 5-formylcytosine (5fC), and 5-carboxylcytosine (5caC) that can be directly converted to cytosine by further enzymatic activities or removed by BER (Figure 1) [21]. DNA methylation may repress transcription by inhibiting the binding of transcriptional activators and/or by facilitating the binding of specific methyl-CpG binding proteins. Proteins possessing methylated DNA binding activity can be divided into three families: methyl-CpG-binding domain-containing MBD proteins (e.g., MeCP2), UHRF proteins/SRA domain family (e.g., UHRF1), and a group of zinc-finger and BTB/POZ domain-containing transcription factors (e.g., Kaiso, ZBTB4, ZBTB38) [22]. These factors bind methylated DNA directly and regulate gene expression by interacting with repressor complexes [23,24], or by participating in the maintenance of DNA methylation [25,26]. Similar to DNMT enzymes, methyl-CpG binding proteins are highly expressed in CNS and are necessary for normal neuronal development and neuronal functions [27,28].

## 3. Lessons from Genome-Wide DNA Methylation Studies

The development of microarray and sequencing-based technologies during the last decades allowed to shed light on DNA methylation changes in HD on a genome-wide scale. These types of studies were performed in cell culture and animal models of the disease as well as on neuronal and non-neuronal patient tissues.

Altered genome-wide cytosine methylation in an HD model was first demonstrated in 2013 by Ng et. al. who applied reduced representation bisulfite sequencing (RRBS) to identify differences in cytosine methylation between mouse striatal neurons expressing full-length *Hdh* (murine *HTT* homolog) with either a pathologic (STHdhQ111) or normal length (STHdhQ7) polyQ domain (Table A1). They identified 21,191 sites with decreased, and 10,413 sites with increased 5mC levels out of 154,239 identified methylated regions. The majority (84%) of the differentially methylated regions (DMR) localized to CpG poor regions, which is characteristic of transcriptional start site (TSS) distal and intergenic sequences. Genes associated with DMRs were enriched for developmental processes, neuron differentiation, neurogenesis, transcription, signaling, and cell adhesion. Comparative analysis of changes in DNA methylation and transcriptional activity in STHdhQ111 cells showed that DMRs located in CpG rich regions, which are characteristic for TSS, negatively correlated with transcriptional activity, while DMRs in CpG poor regions followed a more intricate pattern, including decreased transcription with concurring increased methylation of downstream DMRs, and increased transcription with concurring decreased methylation of downstream DMRs. Based on in silico analysis binding sites of the transcription factors CREB/ATF, AP-1, SOX, and ETS were found enriched in the identified DMRs suggesting that these regions might play a direct role in gene regulation. This was confirmed by ChIP-seq analysis that showed that DMR regions were bound by the AP-1 (Activator protein-1) family transcription factors FRA-2 (Fos-related antigen-2) and JunD, and SOX2. The majority of the binding sites of these transcriptional factors in STHdh cells were CpG poor distal regulatory regions and increased binding of AP-1 or SOX at DMR regions correlated with reduced DNA methylation [29].

In the same year, altered levels of an oxidation product of 5mC, 5hmC, were also published [30]. Although oxidation of 5mC to 5hmC is an intermediate of 5mC demethylation recent data indicates that in animals 5hmC also functions as a stable epigenetic mark [31]. In a study done using the YAC128 mice HD model (Table A1), 5hmC levels in the striatum, cortex, and hippocampus were found to be markedly lower in YAC128 mice compared to age-matched controls, while this effect was not present in the cerebellum and peripheral tissues, suggesting a tissue and brain region-specific effect. Deep sequencing of striatal and cortical samples of 3-months-old, pre-symptomatic YAC128 and control mice showed that 5hmC was enriched in gene bodies (including intragenic CGIs) while depleted in intergenic regions and promoter and intergenic CGIs, revealed distinctive brain region-specific patterns of 5hmC enrichment, and identified disease-specific differentially hydroxymethylated regions (DhMRs). The overwhelming majority of the DhMRs had lower 5hmC levels in YAC128 mice than in controls both in striatal (93%, 698 out of 747) and cortical (90%, 324 out of 362) samples and only 7–10% had increased 5hmC levels. Gene ontology analysis of genes with HD specific DhMRs identified molecular pathways characteristic for both striatal and cortical datasets, like glutamate receptor signaling and CREB signaling, and also striatum specific pathways, such as Wnt/β-catenin signaling, axonal guidance signaling, GABA receptor signaling, Dopamine-DAPRR32 feedback in cAMP signaling, mTOR signaling and synaptic long-term potentiation. These pathways, some of which affect neurogenesis and neuron survival, might be important contributors to early events during HD pathogenesis [30].

The potential role of disturbed DNA methylation during neuronal differentiation was investigated in a human cell culture model by Baronchelli et al. [32]. In this study DNA methylation changes were determined in an induced pluripotent stem cell model (HD-hiPSCs, Table A1) during differentiation towards striatal MSN neurons using Agilent 244 K Human CpG microarray testing 15,200 RefSeq genes. Pluripotent HD-hiPSCs cells showed higher DNA methylation in general than control human embryonic stem cells (hESC). During striatal differentiation increasing hypermethylation was observed, the most dramatic increase occurred between the iPSC stage and the multipotent progenitor cell (rosette) stage while a smaller increase occurred during terminal differentiation. In multipotent progenitor HD cells significantly elevated CpG methylation was observed in promoter regions, gene bodies, intergenic and uncategorized genomic regions compared to corresponding controls. In contrast, in differentiated HD neurons, only the promoter regions showed hypermethylation compared to controls. Differentially hypermethylated regions affected genes involved in axon guidance, neural crest differentiation, or neuroactive ligand–receptor interaction and signaling pathways of neuronal differentiation. Besides these neuronal differentiation-related pathways genes involved in HD-related processes such as cell cycle, insulin, and ubiquitin-mediated proteolysis were also affected by hypermethylation. A large proportion of genes in the overlap of striatal differentiation and HD datasets were related to (interacting with or regulated by) the Jumonji Domain Containing 3 (JMJD3) H3K27 specific histone demethylase providing a link between two different forms of epigenetic regulation, DNA and histone methylation [32].

Genome-wide DNA methylation levels were also investigated in HD patient tissues, both neuronal and non-neuronal. Horvath et al. investigated brain samples of 26 HD cases and 39 controls using Illumina HumanMethylation450K BeadChip array that tests for >450,000 methylation sites within and outside CGIs and covers 99% of RefSeq genes. Age and sex-adjusted genome-wide analysis of CpGs in samples from frontal, parietal, and occipital lobes combined identified 1467 CpGs whose methylation state showed association with HD. Using weighted correlation network analysis the authors defined 54 co-methylation modules common for all three brain regions. From these co-methylation modules, 11 showed a significant correlation with HD status, 5 of these were hypermethylated while 6 were hypomethylated. Genes of HD-associated co-methylation modules are enriched in GO biological process terms including detection of chemical stimulus, nucleic acid metabolic process, and RNA metabolic process [33].

The same group also analyzed DNA methylation changes in blood samples of individuals from the Enroll-HD [34] and Registry-HD [35] observational studies using Illumina Infinium array. By analyzing individual CpG sites genome-wide 33 differentially methylated CpG were identified at the significance level of α = 10^−7^, the most significant hit (cg22982173) fell to the first exon of *HTT* itself. Enrichment analysis of the top 1000 hyper- or hypomethylated CpG sites indicated “RNA binding” as the most enriched functional category among genes affected by hypermethylation, while hypomethylated sites were enriched in genes implicated in polyglutamine diseases by protein interaction networks. Higher methylation of the cg22982173 site in *HTT* also occurs in the nervous system as it was shown to be significantly increased in the right temporal cortex and midbrain of HD patients, and also showed a non-significant increase in the visual, left occipital, and left frontal cortices, and in the cerebellum and caudate nucleus. Hypermethylation of this CpG site (cg22982173) was associated with slower disease progression (motor score, cognitive function assessment) in manifest HD cases, furthermore, hypomethylation of three other CpG sites in or close to PEX14, COX412, and GRIK4 were associated with motor progression. Strikingly, similar to what was observed in the human case DNA methylation of the *Hdh* locus (including both hyper- and hypomethylated CpG sites) was significantly associated with disease state in the striatum and cerebellum of zQ175 knock-in mice (Table A1) and also in blood samples of OVT73 transgenic HD sheep (Table A1) [36].

To investigate tissue-specific transcriptional effects of DNA methylation in HD De Souza et al. analyzed CpG methylation in post-mortem samples of liver and forebrain cortex of HD patients and age-matched controls using Illumina HumanMethylation450K BeadChip array. Comparison of the matched liver–cortical sample pairs identified 38 differentially methylated sites in the *HTT* gene, suggesting that differential CpG methylation might contribute to tissue-specific expression levels of *HTT*. In this study analysis of cortical samples failed to identify any DMRs between HD and control datasets corrected for cell-type proportions. Furthermore, analysis of general methylation levels showed no correlation between methylation state and disease status but showed a significant correlation between methylation state and age of disease onset in the HD cohort [37]. The surprising lack of significant disease state-associated changes in CpG methylation might be due to reduced statistical power as the sample sizes in this study were remarkably smaller than in the Horvath study.

Mutant Htt also seems to alter the epigenetic age of HD patients. The human epigenome shows characteristic changes during the aging process and based on altered DNA methylation patterns an epigenetic age can be calculated [38]. As HD is an age-dependent, progressive disorder several of the above detailed genome-wide DNA methylation studies asked whether mHtt induced pathogenesis alters epigenetic age [33,36,37]. Genome-wide CpG methylation analysis of 475 tissue samples from nine brain regions (Frontal lobe, Occipital lobe, Parietal lobe, Temporal lobe, Caudate nucleus, Cerebellum, Cingulate gyrus, Hippocampus, and Midbrain) indicated that epigenetic age is altered in HD in general, and this effect depended on the clinical severity of the disease: HD cases without the most severe Vonsattel (VS) grade 4 [39] samples showed a significant epigenetic age acceleration effect in the parietal and frontal lobes and the cingulate gyrus, increasing biological age by several years. Surprisingly, however, VS grade 4 samples showed negative age acceleration. Furthermore, epigenetic age acceleration showed a positive correlation with age of motor onset, but a negative correlation with CAG repeat length, the latter effect is most likely related to the decelerated epigenetic aging in VS grade 4 samples that had the longest CAG repeats in the study [33]. Similarly, significant epigenetic age acceleration was shown in manifest HD cases compared to controls by analyzing blood samples from the Enroll-HD and Registry-HD studies, and epigenetic age acceleration showed positively correlated with the progression of motor symptoms [36]. In contrast to the findings above no significant differences in DNA methylation age between human HD and control forebrain cortical samples were found by De Sousa et al. in a smaller sample set [17].

In summary, these analyses demonstrated complex, genome-wide changes in 5mC and 5hmC levels in HD. These include hypomethylation or hypermethylation of specific CpG sites both in CpG poor regions and in CGIs indicating that mHtt induces deregulation, not a generic increase or decrease of DNA methylation. These changes affect genes involved in processes like nucleic acid metabolism, transcription, signal transduction pathways, and neuronal development, among others, and accelerate epigenetic aging. Altered CpG methylation can be observed during neuronal differentiation in mHtt exposed cells, and the *HTT* gene itself is differentially methylated in a genotype and tissue-dependent manner, and its hypermethylation is associated with slower disease progression, most likely by transcriptional downregulation of the diseased allele.

## 4. Targeted Methylation Studies

Besides genome-wide DNA methylation studies targeted analysis of CpG methylation of specific genes relevant in the pathogenesis of HD or other neurodegenerative disorders were also reported.

The ankyrin 1 (ANK1) gene, whose product links integral membrane proteins to the spectrin-actin cytoskeleton, was associated with susceptibility for developing Alzheimer’s disease (AD) and hypermethylation of ANK1 was observed in AD as well as in Parkinson’s disease (PD) [40,41]. Hypermethylation of 4 out of 8 CpG sites in a 118 bp region of ANK1 was also detected in the entorhinal cortex of HD patients but there was no change in ANK1 5mC levels in the cerebellum, superior temporal gyrus, or striatum, the latter of which is primarily affected in HD [41].

Adenosine A_2A_ receptor (A_2A_R, encoded by the *ADORA2A* gene) is a highly expressed G protein-coupled receptor in the striatum, especially in striatopallidal MSN neurons [42], and its level is reduced in the striatum of HD patients and HD mice [43,44,45]. Cytosine methylation was reduced at specific CpG sites of exon m2 of *ADORA2A* in symptomatic (30-weeks-old) R6/1 mice (Table A1), while in symptomatic (12-weeks-old) R6/2 mice (Table A1) the level of 5mC did not change but that of 5hmC was reduced at the same region. Time-course analysis showed that in the R6/1 model altered cytosine modifications at the CGI in the 5′ UTR of *ADORA2A* occurred concomitantly with downregulation of A_2A_R that could be observed beginning from 12 weeks. Specifically, the level of 5hmC in the CGI was reduced in 12-week-old, while the level of 5mC was reduced in 20-week-old mice. Interestingly, in the putamen of HD patients, 5mC was increased, while 5hmC was decreased at the CGI in the 5′ UTR of *ADORA2A*. [45].

Combined transcriptional and methylation analysis drew a link between dysregulated transcriptional factor activity and promoter methylation in the downregulation of Brain-Derived Neurotrophic Factor (BDNF), a key factor downregulated in HD patients and various HD models [46,47]. Transcriptome analysis of Htt-72Q (Table A1) murine primary cortical neurons identified Twist1, a transcriptional factor essential during embryonic development and neoplastic transformations [48], as one of the most robustly upregulated transcriptional factors [8]. Elevated *Twist1* mRNA levels were also observed in patient tissues [49] and the R6/2 and zQ175 mouse models [8]. RNAi-mediated knock-down of *Twist1* resulted in increased viability of Htt-72Q neurons and suppressed neurite degeneration suggesting that *Twist1* overexpression is not a compensatory neuroprotective mechanism but a part of HD pathogenesis and its dysregulation may induce a transcriptional program that contributes to neuronal cell death. Although the upregulation of *Twist1* itself does not seem to be regulated by DNA methylation its knock-down abrogates mHtt induced hypermethylation of the *BDNF* promoter IV and restores BDNF expression to normal levels suggesting that upregulated Twist1 is required for *BDNF* hypermethylation and downregulation [8].

## 5. HD Impacts DNA Methylation Writers, Readers, and Erasers

In light of the above-detailed observations that DNA methylation suffers significant changes in HD, it is not surprising that DNA methyltransferase (DNMT) enzymes are affected by mHtt induced pathology and modulation of their activity can influence pathogenic processes and disease symptoms. Accordingly, DNMT1 and DNMT3B were found to be upregulated in HD patient-derived fibroblasts compared to their wild-type (wt) counterparts. This difference in DNMT levels could be diminished by reprogramming to iPSC cells but differentiation towards HD Neural Stem Cells (NSC) resulted in the upregulation of DNMT3A, TET1, and TET2 expression compared to wt NSC cells [50]. This suggests that alterations of DNA methylation in HD might be the result of dysregulation of DNA methylation reader and eraser enzymes following neuronal differentiation, at least in part. As DNA methyltransferase activity has a profound gene expression regulatory role it might exert influence on HD pathology by indirectly affecting pathologically relevant processes. For example, in a study by Bayer et al. RNAi mediated knock-down of DNMT1 was shown to enhance the retrograde transport of lysosomes and endosomes without affecting anterograde transport. In mHtt expressing cerebellar granule (CB) cells, DNMT1 knock-down was shown to increase the speed of perinuclear mHtt aggregate formation and improve cell survival suggesting that DNMT1 affects HD pathology by modulating aggresome formation via regulation of retrograde transport [51].

The role of DNA methylation in HD pathogenesis is further emphasized by data showing that certain DNMT inhibitors ameliorate HD pathology. An epigenetic drug screen performed in Htt-72Q murine primary cortical neuronal model (Table A1) emphasized the role of DNMT enzymes in HD pathogenesis and presented DNMT inhibitors as potential therapeutic drugs [14]. The screen identified Decitabine (5-aza-2′-deoxycytidine), a nucleoside-analog DNMT inhibitor, as the most effective drug against mHtt induced neurotoxicity that also reduced the amount of mHtt aggregates, and similarly to another nucleoside-analog DNMT inhibitor, 5-fluoro-2′-deoxycytidine (FdCyd), suppressed cell death and neurite degeneration in both Htt-72Q cortical and striatal models. The involvement of DNMT enzymes in HD pathogenesis was investigated directly by RNAi-mediated knockdown of DNMT1 and DNMT3A, both of which resulted in a significant reduction in mHtt induced neurotoxicity. As DNA methylation plays a profound transcriptional regulatory role altered levels or activity of DNMT enzymes may exert their effects by dysregulating genes relevant in HD pathogenesis. Accordingly, Decitabine treatment significantly increased the expression of several key striatal genes that were transcriptionally downregulated in Htt-72Q cells, and experiments with the more stable FdCyd gave similar results in R6/2 mice [14]. One of these genes is *BDNF*, whose exon IV promoter is hypermethylated in the presence of mHtt, as was described above [8]. BDNF transcript levels are reduced in Htt-72Q neurons that coincide with a significant increase of cytosine methylation of 8 CpG sites surrounding the TSS, suggesting that hypermethylation might contribute to the downregulation of *BDNF*. Accordingly, both pharmacological (Decitabine and FdCyd) and molecular (DNMT1 and DNMT3A knock-down) approaches to suppress DNA methyltransferases reversed increased 5mC levels at the *BDNF* exon IV promoter and restored the transcript levels of BDNF in Htt-72Q cells [14]. Similar effects i.e., upregulation of transcription from the *BDNF* promoter IV could be also achieved by suppression of methyl-CpG binding protein 2 (MeCP2), a reader of 5mC marks. MeCP2 was shown to physically interact with Htt in striatal samples of STHdhQ111 mice and cell culture models and this interaction was enhanced by the expanded polyQ domain. MeCP2 binding to the *BDNF* promoter IV was increased in STHdhQ111 cells compared to controls with a parallel decrease in BDNF transcription. BDNF expression was partially restored by RNAi mediated knock-down of MeCP2 suggesting that increased MeCP2 binding might have a direct regulatory role on BDNF transcription in HD [52].

Data showing that genetic or pharmacological manipulation of DNMT activity influences some aspects of HD pathology provokes the question of whether similar effects could be evoked by environmental factors or micronutrients. On the one hand, environmental stressors affecting DNA methylation might be potential risk factors for disease progression, while on the other hand, pathology could be potentially suppressed by dietary supplements affecting CpG methylation levels. Several environmental factors affect DNA methylation, including air pollution, arsenic, tobacco smoke, and nonchemical stressors [53]. Although the effects of these stressors on HD pathology are poorly characterized some of them are known to induce DNA methylation changes on genes involved in HD pathology, for example, the methylation levels of *BDNF*, *DNMT1*, or *TWIST1* were reported to respond to different chemical or nonchemical environmental stressors [53].

DNA methylation can be also influenced by micronutrients like folate (vitamin B9) or cobalamin (vitamin B12) that play a role in the production of the DNMT methyl donor SAM. The precursor of SAM is methionine, which is generated by the transfer of a methyl group from N5-methyltetrahydrofolate to homocysteine by methionine synthase using methyl-cobalamin as a cofactor. N5-methyltetrahydrofolate is synthesized in a multistep enzymatic process in which folic acid is reduced to tetrahydrofolate (THF) that is converted to 5,10-methyleneTHF then reduced to N5-methylTHF by 5,10-methylenetetrahydrofolate reductase (MTHFR) enzyme. Variants of the *MTHFR* gene are known to cause differences in the utilization of folate and influence reactions that are using N5-methylTHF as a methyl donor [54,55]. Thus, normal functioning of this pathway is required for the maintenance of regular levels of SAM, methionine, and homocysteine, the latter of which is neurotoxic itself if accumulated [56]. Folic acid and N5-methylTHF supplementation are beneficial in several medical conditions, most notably during early pregnancy to prevent neural tube defects. Optimal levels of folates are required for neuronal development, memory, and cognitive functions, and folates are also linked to aging-dependent neurodegenerative disorders [57]. For example, in the case of Alzheimer’s disease, the concentration of folate in the cerebrospinal fluid was found to be significantly reduced [58] and 1.25 mg/day folic acid treatment for 6 months was beneficial in a clinical trial [59]. Furthermore, a polymorphism (C677T) of the *MTHFR* gene is a risk factor to develop AD [60]. The information related to the effects of folate in HD are scarce. A study performed on samples of a small group of HD patients indicated that serum levels of vitamin B12 and folic acid are normal in HD [61], while another early study (whose details are not available online) concluded that folic acid treatment was not effective in HD [62]. The potential effects of *MTHFR* variants on HD pathogenesis were investigated but these studies provided contradictory results. Analysis of the relationship of age of disease onset and polymorphisms of homocysteine metabolizing enzymes in 171 HD patients found that patients carrying the *MTHFR A1298C* (rs1801131) variant in homozygous form experienced earlier disease onset [63]. However, a follow-up study analyzing 167 HD patients did not find an association between age of onset and *MTHFR* polymorphisms A1298C and C677T, neither one-by-one nor in combination [64]. Thus, the data currently available about the role of folate in HD is not promising but far too limited to draw a final conclusion.

## 6. Potential Role of DNA Methylation in Transgenerational Effects

DNA methylation acts in concert with other chromatin-modifying mechanisms, such as post-translational modifications of histones, and besides influencing HD pathology they might also exert transgenerational effects conjointly. A study by Jia et al. revealed the interplay of histone (de)acetylation and DNA methylation and implicated these processes in transgenerational epigenetic effects in HD [65]. Treatment with HDACi 4b, a histone deacetylase (HDAC) inhibitor compound that inhibits the activity of HDAC1 and HDAC3, ameliorated disease symptoms of N171-82Q (Table A1) HD mice [66] and also caused transgenerational phenotypical effects, i.e., milder cognitive and motor symptoms in the first filial (F1) generation of treated mice [65]. On the molecular level, significant upregulation of several genes involved in CpG methylation-related epigenetic processes was detected in N171-82Q and R6/2 mice, including *DNMT1*, *DNMT3a*, *Methyl-CpG binding domain protein 3* (*Mbd3*), and *Methyl CpG binding protein 2* (*Mecp2*) [65]. To investigate the interaction between HDAC inhibition and DNA methylation changes the authors analyzed patient-derived HD fibroblasts that exhibit altered 5mC levels at several thousand sites genome-wide. HD fibroblasts showed an altered response to HDAC inhibition as in HD cells larger percentage of genomic CpG sites were hypomethylated after HDACi 4b treatment than in wt cells. One of the differentially DNA methylated genes, *Kdm5d*, that encodes a histone methyltransferase enzyme was also shown to be hypermethylated in sperm of HDACi 4b treated N171-82Q mice and its gene expression levels were elevated in the offspring of these mice, indicating a transgenerational epigenetic effect. Although direct evidence of altered *Kdm5d* DNA methylation in the progeny of HDACi 4b treated mice was not presented this finding suggests a role for DNA methylation in transgenerational effects in HD and suggests an intricate interplay of different epigenetic mechanisms (i.e., histone acetylation and methylation, and DNA methylation) in mHtt induced transcriptional dysregulation [65]. Considering that the HTT locus itself is differentially methylated in HD [36], such transgenerational epigenetic effects might also influence HTT expression and the phenomenon of anticipation (earlier manifestation of the disease in subsequent generations) that is primarily caused by expansion of the disease-causing CAG repeat [67].

## 7. DNA Methylation and Genome Maintenance in HD

Instability of specific repetitive elements of the genome, including trinucleotide repeats and LINE retrotransposons, was observed in mHtt challenged cells. Both of these processes are influenced by altered DNA methylation. Repeat instability leads to, besides other phenomena, the expansion of the CAG trinucleotide repeat in the *HTT* gene itself and consequent disease anticipation.

In HD patient-derived fibroblast cell lines the transcript and protein levels of four DNA repair genes (*APEX1*, *BRCA1*, *RPA1*, and *RPA3*) involved in trinucleotide repeat instability were found to be downregulated. Investigating the epigenetic background of this downregulation the authors found that treatment with 5-Azacytidine (5-AZA), a nucleoside-analog DNMT inhibitor, partially restored the expression levels of at least some of these repair genes in all analyzed cell lines. Accordingly, treatment with 5-AZA inhibited hypermethylation of CpG sites at the *APEX1* promoter in HD fibroblast, and importantly, it also inhibited CAG trinucleotide repeat expansion that could be observed in untreated HD cells [68]. Besides DNMT inhibitor treatment DNA repair gene expression could also be restored by reprogramming of HD fibroblasts to iPSC cells. This effect persisted through differentiation to NSC cells and stabilized CAG repeat length through 20 replication cycles in the iPSC and NCS states. Stabilization of trinucleotide repeats, at least in part, might be the consequence of the restoration of DNMT1 and DNMT3B expression from an upregulated state to wild-type levels in iPSC cells. Expression levels of TET1 and TET2 genes showed no dysregulation in HD fibroblasts and iPSCs, but in the NSC stage TET1 transcript and protein levels were higher in HD cell lines and TET2 transcript and protein levels were higher in one of the cell lines than in wt NSC controls. Knock-down of TET2 resulted in significant downregulation of *RPA1*, and simultaneous knockdown of TET1 and TET2 led to decreased *APEX1* and *RPA3* transcript levels in HD NSC cell lines compared to corresponding wt controls indicating that oxidation of the methyl group of 5mC by TET enzymes play a regulatory role in repair gene expression in HD [50].

Besides trinucleotide repeat instability HD pathology also involves derepression of certain Long Interspersed Nuclear Elements (LINE), a type of non-LTR retrotransposons. In the caudate nucleus but not in non-neuronal (liver) tissues of 10–12-week-old R6/2 HD mice the copy number of LINE-1 (L1) was found to be elevated compared to age-matched wt controls. Transcript and protein levels of L1 ORF1 and ORF2 that were below the detection limit in wt increased significantly in brains of R6/2 mice with concurrent DNA hypomethylation of L1 elements suggesting that disturbed DNA methylation might be responsible for L1 activation. Activation of L1, in turn, might play a role in HD pathology by modulating several survival signaling pathways. This theory is supported by findings showing that similarly to the effects of mHtt expression in R6/2 mice L1 ORF2 overexpression in 293T cells led to downregulation of mTOR1 activity (reduced pS6 and AMPKα levels) and decreased phosphorylation levels of several targets of the pro-survival kinase AKT [69].

## 8. Alternative Base Modifications in Huntington’s Disease

Although cytosine methylation is the most thoroughly characterized and most common DNA modification in the genome [31] other covalent base modifications also exist and might influence gene expression in health and disease. N7-methyl-guanine (7-mG) is a relatively less studied DNA base modification whose presence was demonstrated in DNA from humans not known to be exposed to DNA alkylating agents [70], suggesting that this modification might have an endogenous origin. Altered levels of 7-mG in blood and brain samples of HD patients and transgenic mice were revealed by high-performance liquid chromatography coupled electrochemical detection [71]. Nuclear fractions isolated from brains of 6-to 24-month-old CAG140 KI (Table A1) mice had decreased 7-mG compared to age-matched wt controls. In brain samples of the more drastic R6/2 model, nuclear 7-mG levels were reduced in 12-weeks-old HD mice but, somewhat surprisingly, it was similar to the level of age-matched wt controls in younger animals. Analysis of human post-mortem samples revealed a region and disease grade-specific change in 7-mG levels. In Brodman Area 17 (visual cortex) and the cingulate gyrus of HD patients 7-mG levels were similar to those of controls, while in Brodmann Area 4 (motor cortex) decreased guanine methylation was present in later stages (VS grade 3 and 4) of HD but not in an earlier stage (VS grade 2) [71].

## 9. Possible Application of DNA Methylation as Biomarker in HD

As HD is a monogenic disorder and the disease-causing mutation is known, predictive and diagnostic testing for the disease is possible by simple PCR and is available for nearly three decades [72]. However, accessible molecular biomarkers that are indicative of disease onset or could be used to monitor disease progression or therapeutic effects in trials are still sought for [73]. DNA methylation changes at specific loci have the potential to serve as biomarkers of HD as epigenetic alterations, including changes in CpG methylation, are characteristic of the disease. However, DNA methylation shows tissue-specificity [74], therefore DMRs identified in neuronal tissues are not guaranteed to be suitable pathological markers from peripheral tissue samples. So far only a handful of studies were conducted but these identified a few differentially methylated genes, e.g., *HTT, GRIK4*, or *PEX14*, that might serve as biomarkers of disease progression.

One of the genes that were tested as a potential biomarker is *BDNF*, which is dysregulated in HD with an epigenetic component [10]. In a study performed by Gutierrez et al. DNA methylation of the *BDNF* gene was measured along with BDNF protein levels in blood plasma samples from control, premanifest and manifest HD patients [75]. Akin to other studies [76,77] plasma BDNF protein levels were shown to be similar between controls and HD patients. In contrast, they found significant differences between 5mC levels at the *BDNF* gene. By analyzing 12 CpG dinucleotides at promoter IV of *BDNF* in whole blood samples using bisulfite sequencing they found that four CpG sites were differentially methylated between HD and control samples, three having increased while one having decreased methylation in HD patients. These differences, however, were not striking and did not show a significant correlation with age, CAG repeat number, disease progression (pre-manifest, late pre-manifest, manifest stage), or clinical measures (Mini-Mental State Examination, Unified Huntington’s Disease Rating Scale, Total Functional Capacity, Hospital Anxiety, and Depression Scale) in HD patients [75].

In a genome-wide study by Zadel et al. DNA methylation was investigated in whole blood samples of healthy controls, pre-symptomatic and symptomatic HD patients using Illumina Infinium Human Methylation27 BeadChip microarray testing for the methylation state of 27,578 CpG dinucleotides spanning 14,495 genes [78]. Although the analysis identified several hundred DMRs between these groups by pairwise comparisons, after correction for multiple testing only DMRs of three genes (*CLDN16*, *DDC*, *NXT2*) showed statistically significant differences between pre-symptomatic HD patients and controls, and there were no significant differences in other comparisons (controls vs. symptomatic patients, pre-symptomatic vs. symptomatic patients, controls vs. all HD patients). As the sample sizes in this study were relatively small (15, 9, and 11 individuals in the control, pre-symptomatic, and symptomatic HD groups, respectively) lack of disease stage-specific DNA methylation patterns might have been due to lack of sufficient statistical power [78].

The largest such study to date analyzed DNA methylation changes in blood samples from the Enroll-HD and Registry-HD studies, as described above [36]. 33 differentially methylated CpG sites were identified at the significance level of α = 10^−7^. Hypermethylation of the cg22982173 site in the first exon of *HTT*, which was also observed in neuronal tissues, was the most significant alteration in blood samples of HD patients. Higher methylation of this CpG site (cg22982173) was associated with slower disease progression (motor score, cognitive function assessment) in manifest HD cases, furthermore, hypomethylation of three other CpG sites in or close to *PEX14*, *COX412*, and *GRIK4* were associated with motor progression [36].

## 10. Conclusions

**Figure 2:**
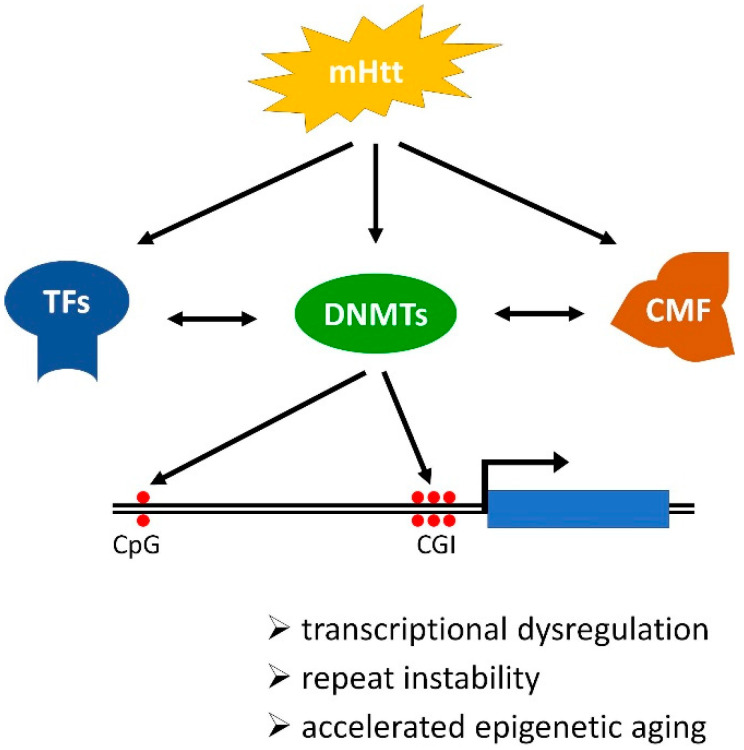
Schematic illustration of the mechanism and consequences of DNA methylation changes in Huntington’s disease (HD). Mutant Huntingtin (mHtt) directly or indirectly modulates the activities of transcriptional factors (TF), DNA methyltransferases (DNMT), and chromatin-modifying factors (CMF) that act in concert to alter the chromatin landscape, including DNA methylation. In HD altered DNA methylation occurs genome-wide and affects both transcriptional start site (TSS) proximal CpG islands (CGI) and intergenic CpG sites thereby influencing transcription regulation, stability of trinucleotide repeats, and mobile genetic elements, and epigenetic age.

HD is characterized by multifaceted pathogenesis that includes alterations of the chromatin landscape. mHtt provokes changes in the level or activity of transcriptional factors, chromatin-modifying factors, and enzymes involved in CpG methylation (DNMTs) and demethylation (TETs) directly or indirectly. The concerted action of these factors leads to complex changes in DNA methylation in HD, that include simultaneous hyper- or hypomethylation of specific CpG sites residing in different parts of the genome including both CpG poor intergenic regions and TSS proximal CGIs. These changes in DNA methylation contribute to HD pathology by affecting DNA-dependent processes and thereby accelerating epigenetic aging, destabilizing trinucleotide repeats and mobile genetic elements, and modifying the transcriptional activity of genes (Figure 2). Certain differentially methylated CpG sites, for example, cg22982173 in *HTT* itself, might prove to be useful biomarkers, and the fact that some DNMT inhibitors that are FDA approved for the treatment of other ailments ameliorate some aspects of HD pathology are especially encouraging. It is important to remember, however, that interfering with epigenetic mechanism might prove to be a double-edged sword, that on the one hand improves some aspects of HD pathology (e.g., reduced trinucleotide repeat instability and improved BDNF levels), while on the other hand makes other aspects worse (e.g., increased instability of LINE-1 elements and hypomethylation of *HTT* that correlates with disease progression). Thus, although modulation of DNA methylation holds the promise of therapeutic use better understanding of the involved and affected processes is needed to be able to estimate the risks and benefits of potential treatment options and achieve planned outcomes.

## Data Availability

Not applicable.

## Appendix A

**Table A1:** List of Huntington’s disease models used in the reviewed studies.

Model	Description	Reference
CAG140 KI	knock-in murine model expressing full-length *Hdh* gene with exon1 from human *HTT* with 140 CAG repeats	[79]
HD-hiPSCs	human-induced pluripotent stem cells derived from HD patients differentiated towards medium spiny neurons	[32]
Htt-72Q	murine primary cortical neuron model expressing *HTT* exon1 with 72 CAG repeats	[8]
OVT73	transgenic sheep model expressing full-length human *HTT* cDNA with 73 CAG/CAA repeats under the control of the human promoter	[80]
R6/1	murine model transgenic for the 5′ end of the *HTT* gene carrying 116 CAG repeats	[81]
R6/2	murine model transgenic for the 5′ end of the *HTT* gene carrying 144 CAG repeats	[81]
N171-82Q	Transgenic murine model expressing N-terminal truncated human *HTT* cDNA with 82 CAG repeats under the influence of mouse prion protein promoter	[82]
STHdhQ111	cell culture model derived from knock-in mouse embryos expressing full-length *Hdh* ^1^ with 111 CAG repeats	[83]
YAC128	yeast artificial chromosome transgenic murine model expressing full-length *HTT* transgene with 128 CAG repeats	[84]
zQ175	knock-in murine model expressing full-length *Hdh* gene with exon1 from human *HTT* with 188 CAG repeats, derived from CAG140 KI	[85]

^1^ Murine homolog of human *HTT*.
